# HIV, Hepatitis B and C among people who inject drugs: high prevalence of HIV and Hepatitis C RNA positive infections observed in Delhi, India

**DOI:** 10.1186/s12889-015-2003-z

**Published:** 2015-07-30

**Authors:** Lopamudra Ray Saraswati, Avina Sarna, Mary Philip Sebastian, Vartika Sharma, Ira Madan, Ibou Thior, Julie Pulerwitz, Waimar Tun

**Affiliations:** Population Council, Zone 5A, India Habitat Centre, New Delhi, 110003 India; Sahara Center for Residential Care and Rehabilitation, New Delhi, India; ARISE Project, PATH, Washington, DC USA; Population Council, Washington, DC USA

**Keywords:** HIV, Hepatitis B, Hepatitis C, HIV-HCV co-infection, People Who Inject Drugs (PWID), India

## Abstract

**Background:**

India has large PWID (persons who inject drugs) population estimated at 177,000. PWIDs are at high risk for HIV, Hepatitis B (HBV) and Hepatitis C (HCV) infections. We report the prevalence of HIV, HBV and HCV infections and correlates of HIV-HCV co-infection among male PWIDs in Delhi.

**Methods:**

3748 male PWIDs were recruited for a longitudinal HIV incidence study. Participants were tested for HBV and HCV infections at their first follow-up visit (FV1) using serum HBV-surface antigen, and HCV-antibody tests followed by HCV RNA PCR, respectively. All PWIDs who were HIV-negative at enrollment, were re-tested for HIV at FV1. Multinomial logistic regression was employed to identify predictors of HIV, HCV and HIV-HCV co-infection.

**Results:**

Overall prevalence of HIV, HBV and HCV among 2,292 participants tested at FV1 was 25.9 %, 9.7 % and 53.7 %, respectively. 6.4 % of the participants had HIV mono-infection, 34.1 % had HCV mono-infection, and 19.6 % had HIV-HCV co-infection. 26 % of HIV-positive participants without HCV were HBsAg positive.

In the regression model, having practiced at least one risky injection in the past month (relative risk ratio (RRR): 1.38; 95 % CI: 1.01-1.89) and not knowing his own HIV status (RRR: 1.65, 95 % CI: 1.25-2.17) were independent predictors for HIV-HCV co-infection. Longer duration of drug injections was associated with a higher likelihood of HCV mono-infection (2–5 years RRR: 2.13; 6–10 years RRR: 2.74; ≥11 years RRR: 3.14) and HIV-HCV co-infection (2–5 years RRR: 5.14; 6–10 years RRR: 8.53; >11 years RRR: 8.03). Higher frequency of injection days/month was associated with a higher likelihood of HCV mono-infection (≤10 days/month RRR: 1.61; 11–20 days/month RRR: 3.15; 21–30 days/month RRR: 3.47) and HIV-HCV co-infections (≤10 days/month RRR: 2.26; 11–20 days/month RRR: 3.46; 21–30 days/month RRR: 4.83).

**Conclusions:**

We report a high prevalence of HIV, HCV and HIV-HCV co-infection among male PWIDs in Delhi. A tenth of the participants were HBsAg positive. Targeted Intervention programs should make HBV/HCV testing, prevention and care more accessible for PWIDs.

## Background

People who inject drugs (PWIDs) are at high risk for blood-borne infections that include HIV, hepatitis B (HBV) and C virus (HCV). HCV has been identified as the most common viral infection affecting PWIDs [1] and HCV is estimated to be 10 times more infectious than HIV, per unit of blood required for transmission [1]. Chronic HCV infection is associated with chronic liver disease including cirrhosis and end-stage liver disease [2, 3]; about 80 % of individuals exposed to HCV develop chronic infection and 3-11 % with chronic HCV develop liver cirrhosis within 20 years [4]. HIV and HCV co-infection adversely affect the course and prognosis of both conditions [5–9]. In the case of HBV, 5 % of adults exposed to the virus develop chronic HBV infection, and cirrhosis and death because of hepatocellular carcinoma are important complications of chronic HBV infection [10, 11].

The HIV epidemic in India is concentrated among sex workers, people who inject drugs (PWIDs), and men who have sex with men – often called ‘most-at-risk-populations’ (MARPs) - and among these groups PWIDs have the second highest HIV prevalence in the country (7.14 %) [12]. India has an estimated 177,000 PWIDs [12]. The PWID population has been largely studied in the high HIV prevalence states in north-eastern and southern parts of the country, where HIV (25.4 -59.6 %), HBV (10 %) and HCV (54.5-90.4 %) prevalence has been reported [9, 13–15]. Although there is emerging evidence of PWID presence in low HIV prevalence states of the country (the north-western part), only a few studies have documented HIV (29-37 %), HBV (40 %) and HCV (36-49 %) prevalence in these states [16–18]. The PWID populations in the northeastern, southern, and western parts of the country differ significantly on socio-demographic characteristics, drug use patterns, and awareness of HIV and hepatitis B and C infections [19] highlighting the need for more epidemiological evidence on the prevalence and correlates of hepatitis in the PWID population. In this paper we report on the prevalence of HIV and Hepatitis B and C infections in a large cohort of male PWIDs from Delhi. Further we characterize the correlates of HIV and Hepatitis C co-infection in this population.

## Methods

### Study setting

The Population Council, in collaboration with PATH’s Arise - *Enhancing HIV Prevention Programs for At***-***Risk Populations*, initiated a longitudinal cohort study, at five Drop-in Centers (DICs) run by Sahara Centre for Rehabilitation and Residential Care in Delhi, to examine HIV incidence and behavior change among PWIDs, before and after the provision of comprehensive HIV prevention interventions that follow WHO/UNAIDS/UNODC guidelines. The study entailed three rounds of data collection: baseline, follow-up visit 1 (FV1) and follow-up visit 2 (FV2); the median follow-up time between baseline and FV1 was 9.8 months (IQR: 8.2-11.1 months), and between FV1 and FV2 was 12.0 months (IQR: 10.7-13.2 months). HIV testing was undertaken at baseline to determine the HIV status of the cohort [20]. At FV1, all participants underwent testing for hepatitis B and C; HIV-negative participants also underwent a second round of HIV testing at FV1. As funds were available only for one round of hepatitis B and C testing, no tests were conducted at FV2. In this paper we present data from the FV1. Harm reduction services were initiated after completion of FV1.

Participants were recruited through peer-referral and targeted outreach by outreach workers (ORW). Prior to study initiation, a mapping exercise was conducted to identify all hotspots where PWIDs congregated in central, east, north-east and north-west districts of Delhi where the five Sahara Drop-in Centers (DIC) were located. The peer-referral recruitment process was initiated using eleven ‘seed’ participants (recruiters) across the five study sites; each ‘seed’ participant was provided with five recruitment coupons to give out to PWIDs in their network. New recruits were linked to the recruiter through unique ID numbers; each new recruit received five coupons to recruit other PWIDs and the recruiter received a ‘food coupon’ for each new PWID s/he brought into the study. Food coupons were exchanged for food at selected restaurant outlets. For recruitment using targeted outreach, ORWs visited hotspots to invite PWIDs to participate in the study and willing PWIDs were directed to the study site with an ORW coupon. All PWIDs who had a peer-referral recruitment coupon were considered recruited through peer-referral while those who came to the site with an ORW coupon were listed as recruited by ORWs. Walk-in clients who did not have either a peer-referral or ORW coupon were also permitted to enroll. All PWIDs who enrolled in the study, including walk-in clients and those recruited by ORWs, received five recruitment coupons each to recruit other PWIDs. Every hotspot was covered during the recruitment phase and recruitment continued till no new PWIDs could be found [21]. To be eligible, participants had to be 18 years of age or older, current IDU defined as injecting at least once in the last 3 months and residing in Delhi. All participants provided written informed consent. All participants received Rs 40 (approximately 80 US cents) for participating in the behavioral survey.

To detect duplicate registrations at recruitment, we used a web-based, live data-base accessible by all five DICs which allowed the DIC site manager to verify new clients against already registered clients. Registration procedures included the recording of photographs and the following identifiers: name, age, gender, marital status, height, weight and fore-arm length – participants presenting with similar identifiers were identified by the computer program for further scrutiny [21].

At FV1, ORWs contacted participants in the community to return for their follow-up visit. Returning participants completed FV1 data collection which included laboratory testing and a behavioral survey. As very few female PWIDs (n = 26) were recruited in the study, they have been excluded from the analysis.

The study was approved by the National AIDS Control Organization (NACO) Ethics Committee in Delhi, the Research Ethics Committee of PATH in Seattle, USA and the Institutional Review Board of the Population Council in New York. All participants provided written informed consent for participating in the behavioral survey and collection of blood samples.

### Laboratory testing

HIV sero-status at baseline was determined using NACO HIV testing guidelines via rapid tests: HIV-negative status was based on a single highly sensitive rapid test (DetermineTM HIV-1/2; Inverness Medical Japan Co Ltd., Japan) and a positive result on two additional confirmatory rapid tests (SignalTM HIV-1/2; Span Diagnostics, Surat India and TridotTM; Biomed Industries, Himachal Pradesh, India). At FV1, all HIV-negative male PWIDs were tested using a fourth generation Antigen-Antibody test (ARCHITECT HIV Ag/Ab Combo; Abbott Laboratories, Illinois, USA) followed by a confirmatory Western Blot Assay (HIV Blot 2.2 Western blot assay; MP Biomedicals Asia Pacific Limited, Singapore). For participants with a negative or indeterminate result on the Western blot test at FV1, a plasma HIV RNA PCR test (COBAS® Ampliprep/COBAS® TaqMan® HIV-1 Test; Roche Diagnostics, Tokyo, Japan) was conducted on the FV1 plasma sample to identify any window period infection at FV1.

Hepatitis C antibodies were detected using CMIA/Electrochemiluminescence test (ARCHITECT Anti-HCV Reagent Kit; Abbott Laboratories, Illinois, USA). All participants with a positive HCV antibody test underwent HCV RNA PCR testing (COBAS® AmpliPrep/COBAS® TaqMan® HCV Tests; Roche Diagnostics, Tokyo, Japan); participants positive for HCV RNA were labelled HCV positive. We have considered HCV RNA positivity as the marker for HCV prevalence so as to accurately identify active HCV infection and, therefore, PWIDs who have the potential to transmit infection to others. HBV infection was detected using serum hepatitis B surface antigen test (ARCHITECT HBsAg Reagent Kit; Abbott Laboratories, Illinois, USA).

### Statistical analysis

All quantitative analyses were conducted using STATA version 11.2 (Stata Corp, College Station, Texas, USA). Participants were categorized into four groups with regard to their HIV and HCV status: (a) Sero-negative (HIV-, HCV-), (b) HIV mono-infection (HIV+, HCV-), (c) HCV mono-infection (HIV-, HCV+), and (d) HIV & HCV co-infection (HIV+, HCV+). Comparisons were made between sero-negative participants (HIV- and HCV-) and the different infection groups (HIV mono-infection, HCV mono-infection, HIV & HCV co-infection) using Chi-squared test for categorical variables and Mann–Whitney U test for continuous variables. Prevalence of HBV was reported in these four infection categories.

We used a multinomial logistic regression model to predict the relative risk ratios for HIV mono-infection, HCV mono-infection and HIV-HCV co-infection as compared to the sero-negative PWIDs (reference category). Variables that were found to be significant in at least one of the infection categories from the bivariate analysis from Table 1, were included in the regression model (Table 2). Regional origin was classified into three categories – Delhi, three adjacent states of Delhi (includes neighboring states Uttar Pradesh, Haryana and Rajasthan), and other states. HIV knowledge was assessed using a 6-item index comprising knowledge that HIV transmission can be prevented by (i) correct and consistent use of condoms for sex and (ii) having a monogamous uninfected sexual partner; that (iii) sharing of needles/syringes increase the risk of HIV transmission, that (iv-v) HIV infection cannot spread from mosquito bites or from sharing food; and that (vi) healthy-looking people can be infected with HIV. Comprehensive knowledge was considered to be present if participants responded correctly on all 6 items. Prior knowledge of HIV status was also included.

**Table 1:** Socio-demographic characteristics, risky injection and sexual behaviors and service utilization by male PWID by HIV, HCV and HIV-HCV co-infection status, Delhi (2012)

	Total	Sero-negative	HIV mono-infected		HCV mono-infected		HIV & HCV co-infected	
		% (n)	% (n)	p-value^1^	% (n)	p-value^1^	% (n)	*p*-value^1^
Number of PWIDs	2,292	100.0 (916)	100.0 (146)		100.0 (781)		100.0 (449)	
Prevalence of sero-infections (%)		40.0	6.4		34.1		19.6	
Socio-demographic characteristics								
Age (median, IQR)	29 (23, 37)	28 (23, 38.5)	26 (22, 35)	0.016 ^2^	29 (24, 38)	0.873 ^2^	30 (24, 35)	0.772^2^
Education				0.371		0.972		
Illiterate	47.6 (1090)	46.8 (429)	48.6 (71)		46.3 (361)		51 (229)	2.8; 0.246
Class 1-6	27.6 (632)	27.4 (251)	30.8 (45)		27.6 (215)		27 (121)	
7 or higher	24.8 (569)	25.8 (236)	20.6 (30)		26.2 (204)		22.1 (99)	
Marital status				0.112		0.545		0.043
Married/cohabiting	34.7 (796)	36.7 (336)	30.1 (44)		34.8 (272)		32.1 (144)	
Never married	52.9 (1213)	52.6 (482)	54.1 (79)		53 (414)		53 (238)	
Divorced/widowed	12.4 (283)	10.7 (98)	15.8 (23)		12.2 (95)		14.9 (67)	
Religion				0.921		<0.001		0.609
Hindu	62 (1418)	58.5 (535)	58.9 (86)		69.5 (541)		57 (256)	
Non-Hindu	38 (870)	41.5 (380)	41.1 (60)		30.5 (237)		43 (193)	
Regional origin				0.003		0.010		0.010
Delhi	25.4 (580)	25.4 (232)	24.7 (36)		25.4 (198)		25.4 (114)	
3 states adjacent to Delhi	44.4 (1016)	39.9 (365)	53.4 (78)		46.2 (360)		47.4 (213)	
Others	30.2 (692)	34.7 (317)	21.9 (32)		28.4 (221)		27.2 (122)	
Duration of residence in Delhi (median, IQR)	22 (15, 30)	22 (15, 30)	20 (12, 29)	0.142 ^2^	23 (16, 30)	0.124 ^2^	23 (15, 30)	0.208 ^2^
Accommodation				0.183		0.879		0.801
Living at home with family	44 (1009)	44.4 (407)	39.7 (58)		44.4 (347)		43.9 (197)	
Living In rented/paying guest	17.5 (400)	17.6 (161)	14.4 (21)		18.4 (144)		16.5 (74)	
Living on the street	38.5 (883)	38 (348)	45.9 (67)		37.1 (290)		39.6 (178)	
Comprehensive HIV knowledge				0.190		0.908		0.048
No/incomplete knowledge	63.2 (1448)	64.5 (591)	58.9 (86)		64.8 (506)		59 (265)	
Complete knowledge	36.8 (844)	35.5 (325)	41.1 (60)		35.2 (275)		41 (184)	
Alcohol use				0.046		0.859		0.009
Never	54.4 (1246)	51.9 (475)	61 (89)		53.1 (414)		59.8 (268)	
2 times/week or less	35.6 (815)	37.1 (339)	33.6 (49)		35.8 (279)		33 (148)	
3 times a week or more	10 (228)	11 (101)	5.5 (8)		11.2 (87)		7.1 (32)	
Sex with regular or paid/non-regular female partner in last 3 months				0.008		0.110		<0.001
No sex	63.5 (1455)	57.6 (528)	71.2 (104)		62.6 (489)		74.4 (334)	
Safe sex	11 (251)	12.3 (113)	8.2 (12)		10.5 (82)		9.8 (44)	
Unsafe sex	25.6 (586)	30 (275)	20.6 (30)		26.9 (210)		15.8 (71)	
Service utilization in the last 3 months								
Accessed NSP services				<0.001		<0.001		<0.001
No	55.5 (1271)	70.4 (645)	43.2 (63)		48.9 (382)		40.3 (181)	
Yes	44.6 (1021)	29.6 (271)	56.9 (83)		51.1 (399)		59.7 (268)	
Accessed detox/rehab services				0.696		0.270		0.148
No	94.1 (2157)	93.3 (855)	92.5 (135)		94.6 (739)		95.3 (428)	
Yes	5.9 (135)	6.7 (61)	7.5 (11)		5.4 (42)		4.7 (21)	
Accessed OST services				0.847		0.643		0.583
No	89.3 (2046)	89.2 (817)	89.7 (131)		89.9 (702)		88.2 (396)	
Yes	10.7 (246)	10.8 (99)	10.3 (15)		10.1 (79)		11.8 (53)	
Duration and frequency of injection drug use								
Time since first injection				<0.001		<0.001		<0.001
1 year or less	13.2 (299)	22.5 (204)	9.7 (14)		8.6 (66)		3.4 (15)	
2-5 years	53.8 (1221)	52.7 (478)	54.5 (79)		55.1 (425)		53.6 (239)	
6-10 years	21.6 (490)	15.9 (144)	27.6 (40)		22.5 (174)		29.6 (132)	
11 years or more	11.5 (260)	8.9 (81)	8.3 (12)		13.9 (107)		13.5 (60)	
Number of days injected in the past month				<0.001		<0.001		<0.001
Not injected	26.5 (606)	44.4 (406)	19.2 (28)		15.7 (122)		11.1 (50)	
1-10 days	20.5 (470)	23.5 (215)	16.4 (24)		18.2 (142)		19.8 (89)	
11-20 days	16.7 (382)	11.4 (104)	17.1 (25)		21.2 (165)		19.6 (88)	
21-30 days	36.3 (830)	20.7 (189)	47.3 (69)		44.9 (350)		49.4 (222)	
Risky injection behavior in the past 1 month				<0.001		<0.001		<0.001
No risk	47.6 (1090)	63.7 (583)	39 (57)		37.6 (294)		34.7 (156)	
At least one risky injection	52.4 (1202)	36.4 (333)	61 (89)		62.4 (487)		65.3 (293)	
Prior HIV test								
Prior knowledge of own HIV status				0.001		0.778		<0.001
Know the result	71.4 (1631)	74.5 (681)	61 (89)		73.9 (575)		64.1 (286)	
Not tested/ Result not known	28.6 (653)	25.5 (233)	39 (57)		26.1 (203)		35.9 (160)	

^1^ Chi-squared test, unless specified, for each infection category compared with sero-negative participants

^2^ Mann–Whitney U test for each infection category compared with sero-negative participants

NSP: Needle Syringe Program; OST: Oral Substitution Therapy

**Table 2:** Multinomial Logistic Regression analysis to determine predictors of HIV, HCV and HIV-HCV prevalence among Male PWID in Delhi (2012)^1, 2^

	HIV mono-infection	HCV mono-infection	HIV & HCV co-infection
	RRR (95 % CI); p-value	RRR (95 % CI); p-value	RRR (95 % CI); p-value
Comprehensive HIV knowledge			
No comprehensive knowledge	1.00	1.00	1.00
Having comprehensive knowledge	1.50 (1.03 - 2.20); 0.037	1.08 (0.86 - 1.34); 0.523	1.47 (1.13 - 1.91); 0.004
Alcohol use			
Never	1.00	1.00	1.00
≤2 times/week	0.85 (0.57 - 1.27); 0.432	0.96 (0.77 - 1.21); 0.759	0.88 (0.67 - 1.16); 0.363
≥3 times a week	0.62 (0.28 - 1.36); 0.236	1.27 (0.89 - 1.81); 0.18	0.85 (0.53 - 1.36); 0.486
Sex with any female partner in last 3 months^3^			
No sex	1.00	1.00	1.00
Safe sex	0.55 (0.29 - 1.06); 0.076	0.79 (0.56 - 1.11); 0.17	0.62 (0.41 - 0.95); 0.026
Unsafe sex	0.60 (0.37 - 0.99); 0.046	0.93 (0.71 - 1.21); 0.567	0.40 (0.28 - 0.57); <0.001
Accessed NSP services last 3 months			
No	1.00	1.00	1.00
Yes	1.91 (1.26 - 2.89); 0.002	1.37 (1.08 - 1.73); 0.009	1.74 (1.32 - 2.30); <0.001
Duration of injecting drug use			
≤1 year	1.00	1.00	1.00
2-5 years	1.90 (1.02 - 3.51); 0.042	2.13 (1.53 - 2.95); <0.001	5.14 (2.91 - 9.09); <0.001
6-10 years	3.01 (1.52 - 5.97); 0.002	2.74 (1.86 - 4.03); <0.001	8.53 (4.65 - 15.64); <0.001
11 years of more	2.07 (0.86 - 5.00); 0.106	3.14 (2.00 - 4.93); <0.001	8.03 (4.09 - 15.76); <0.001
Frequency of injection during the past month			
Never	1.00	1.00	1.00
1-10 days	0.93 (0.47 - 1.83); 0.831	1.61 (1.14 - 2.29); 0.007	2.26 (1.43 - 3.57); <0.001
11-20 days	1.57 (0.76 - 3.24); 0.226	3.15 (2.12 - 4.66); <0.001	3.46 (2.09 - 5.74); <0.001
21-30 days	2.47 (1.30 - 4.69); 0.006	3.47 (2.43 - 4.95); <0.001	4.83 (3.06 - 7.61); <0.001
Risky injection practice in last one month			
Never	1.00	1.00	1.00
At least one risky injection	1.40 (0.87 - 2.26); 0.171	1.39 (1.06 - 1.83); 0.016	1.38 (1.01 - 1.89); 0.045
Prior knowledge of own HIV status^4^			
Know the result	1.00	1.00	1.00
Not tested/Don't know the result	1.92 (1.31 - 2.82); 0.001	0.97 (0.76 - 1.23); 0.813	1.65 (1.25 - 2.17); <0.001

^1^Reference category of the dependent variable in the multinomial regression model is sero-negative participants (n = 916) compared to participants with HIV mono-infection (n = 146), HCV mono-infection (n = 781) and HIV & HCV co-infection (n = 449)

^2^Controlled for age, religion, marital status and regional origin

^3^Any female partner includes regular, non-regular or paid

^4^At baseline

Risky injection behavior was derived from a 5-item index of various injection practices in the last month:

(i) using needles or syringes previously used by someone else, (ii) back/ front loading/splitting of drugs, (iii) receiving an injection with a syringe filled by someone else, (iv) drawing up drugs from a common container, and (v) sharing vial, cooker, container, cotton, filter or water with other injecting partners. A regular sex partner was defined as a spouse or live-in partner. A paid partner was someone the respondent had sex with in exchange for money, gifts or drugs. A non-regular partner was someone the respondent was not living with or paid for sex. Safe sex was defined as use of condoms every time the respondent had sex. Unsafe sex was defined as inconsistent or no use of condoms.

## Results

**Fig. 1:**
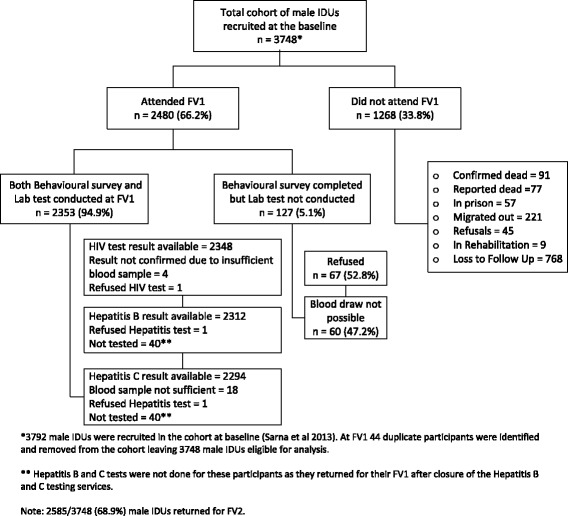
Follow-up and Laboratory testing of Male PWIDs in the baseline full Cohort

A total of 3792 male PWIDs were recruited at baseline [20]. Between baseline and FV1, 44 duplicate clients were identified and removed from the database. Of the remaining male PWIDs 66.2 % (2480/3748) participants returned for their FV1; the behavioral survey and blood tests were conducted for 2353 participants (Fig. 1). As shown in Fig. 1, HIV test results were available for 2348 participants, hepatitis B for 2312 participants and hepatitis C test results for 2294 participants. Our data analysis includes the 2292 male PWIDs for whom we have HIV, HBV and HCV test results.

The median age of study participants was 29 years (IQR: 23–37). Most of them were illiterate (48 %), never married (53 %), and of the Hindu faith (62 %). All participants resided in Delhi, however, 44 % were originally from the three adjacent states of Delhi and 33 % from other states (Table 1). While most of the participants were home-based (44 %), a sizeable proportion lived on the streets (39 %). HIV knowledge was low (37 %); 54 % reported never having consumed alcohol and 63 % had been sexually inactive in the last 3 months. Only 9.1 % of participants had ever heard of hepatitis B and 3.1 % had heard of hepatitis C infections (data not shown). A third had been injecting for more than 6 years. Unsafe injection practices were common: 26.6 % reported injecting with a used needle in the last one month, 32 % had shared injecting equipment, 15.4 % had received a prefilled injection from another PWID, 18.3 % had injected drugs using front/back loading and splitting and 48.1 % had drawn drugs from a common container (data not shown); overall 52.4 % participants reported risky injection behavior in the last one month (Table 1). 44.5 % had accessed needle/syringe exchange/distribution services in the last 3 months, while uptake of oral substitution therapy (OST) and rehabilitation services was low (Table 1). The vast majority of participants (69.2 %) reported having injected opioids (heroin/brown sugar/smack/synthetic opioids) and 73.4 % had injected tranquilizers/antihistaminics in the last one month; a few reported injecting cocaine (0.5 %) and amphetamines (3.1 %). Participants typically injected a cocktail of two or three drugs consisting of one opioid and tranquilizers and/or antihistaminics (data not shown).

### Prevalence of HIV, HCV and HIV-HCV co-infection

HCV antibody tests were positive for 70.9 % (1626/2294) of the participants; of those 75.7 % (1230/1626) participants tested positive on HCV RNA PCR test, and were considered HCV positive; overall 53.7 % (1230/2292) of participants were HCV RNA positive. Overall 6.4 % (n = 146) participants had HIV mono-infection, 34.1 % (n = 781) had HCV mono-infection and 19.6 % (n = 449) HIV & HCV co-infection while 39.9 % were sero-negative for HIV or HCV. HIV prevalence was 25.9 % (595/2292); this includes the 109 new seroconversions among HIV-negative PWIDs documented between baseline and FV1. Table 1 highlights the differences between the various infection groups compared to uninfected participants. On bivariate analysis, there were no significant differences between various infection groups and uninfected participants on age, education, duration of residence in Delhi or accommodation. All three infection groups were more likely to have migrated from the 3 states adjacent to Delhi. HIV & HCV co-infected participants were more likely to have comprehensive HIV knowledge, report non-consumption of alcohol, be divorced/widowed and not sexually active in the last 3 months than sero-negative participants. HIV mono-infected participants were also more likely to be not sexually active. All three infection groups were more likely to have been injecting for longer periods (>2 years) and on more days every month, to have higher scores on the risky injection index and to have accessed NSP services than uninfected participants (Table 1).

### Prevalence of HBV, HIV-HBV and HBV-HCV co-infections

Hepatitis B surface antigen was detected in 9.7 % (222/2292) participants. HBV prevalence was 6.7 % (52/781) among the HCV mono-infected and 8.7 % (39/449) among the HIV-HCV co-infected participants. The highest HBV prevalence (26 %; 38/146) was found among those who were HIV positive but HCV negative. The prevalence of HBV mono-infection was 10.2 % (93/916).

### Results from multivariate multinomial logistic regression

Results of multinomial logistic regression analysis to determine correlates of HIV mono-infection, HCV mono-infection, and HIV & HCV co-infection with reference to uninfected participants, after controlling for age, marital status, religion and regional origin, are presented in Table 2. Participants who had not undertaken HIV testing or did not know of their HIV-status prior to the study were at higher risk of HIV mono-infection (RRR: 1.92) and HIV-HCV co-infection (RRR: 1.65). Longer duration of drug injections was associated with a higher likelihood of HCV mono-infection with a dose response effect (2–5 years RRR: 2.13; 6–10 years RRR: 2.74; >11 years RRR: 3.14 years) and HIV-HCV co-infection (2–5 years RRR: 5.14; 6–10 years RRR: 8.53; >11 years RRR: 8.03). A higher frequency of injection days/month was associated with a higher likelihood, with a dose–response effect, for HCV mono-infection (≤10 days/month RRR: 1.61; 11–20 days/month RRR: 3.15; 21–30 days/month RRR: 3.47) and HIV-HCV co-infections (≤10 days/month RRR: 2.26; 11–20 days/month RRR: 3.46; 21–30 days/month RRR: 4.83). Risky injection behavior in the last one month was an independent predictor of HCV mono-infection (RRR: 1.39) and HIV-HCV co-infection (RRR: 1.38).

Having comprehensive HIV knowledge was associated with a higher risk of HIV mono-infection (RRR: 1.5) and HIV-HCV co-infection (RRR: 1.47). Further, a higher likelihood of all three categories of infection was observed among participants who had accessed needle syringe program services in the last three months (HIV mono-infection RRR: 1.91; HCV mono-infection RRR: 1.37; HIV-HCV co-infection RRR: 1.74). These results are unexpected and need to be interpreted with caution. On further analysis, it was observed that (a) comprehensive HIV knowledge increased with longer duration of injection use (<1 year: 27 %; 2–5 years: 36 %; 6–10 years: 41 %; >11 years: 45 %; p < 0.001); (b) the use of needle syringe program services increased with longer duration of injection drug use (<1 year: 27 %; 2–5 years: 44 %; 6–10 years: 54 %; >11 years: 53 %; p < 0.001) and frequency of injections in the last 1 month (no injection: 8 %; 1–10 days: 48 %; 11–20 days: 63 %; 21–30 days: 62 %; p < 0.001); and (c) among PWIDs who accessed NSP services, risky injection practices increased with greater frequency of injections in last 1 month (no injections: 1.92 %; 1–10 days: 65.9 %; 11–20 days: 76.9 %; 21–30 days: 79.1 %; p < 0.001). However, comprehensive HIV knowledge was not associated with use of needle syringe programs services: 44 % of participants with comprehensive knowledge and 46 % without comprehensive knowledge had accessed needle syringe program services in the last 3 months (p = 0.26). Public sector HIV prevention services delivered through Targeted Intervention (TI) programs for PWIDs in Delhi have been in existence for several years. PWIDs with longer duration of injections are more likely to be a part of the PWID community for a longer period of time, and therefore, more likely to be covered by NSP and IEC services. PWIDs with a higher frequency of injections are more likely to seek NSP services due to a greater need for clean needles. Therefore, PWIDs with longer duration of injecting and greater frequency of injections remain most at risk for new infections despite accessing NSP services as they may not obtain a sufficient supply of clean needles to meet their need, and continue to engage in unsafe injection practices as shown. Needle syringe distribution as part of our study was initiated only after completion of FV1.

Any sex (safe sex RRR: 0.62 and unsafe sex RRR: 0.40) compared to no sex was associated with a lower likelihood of HIV-HCV co-infection; unsafe sex (RRR: 0.60) was also associated with a lower likelihood of HIV mono-infection. In our cohort, only 37 % of the participants were sexually active; sexual activity decreased with duration of injection drug use (≤1 year: 45 %; 2–5 years: 36 %; 6–10 years: 36 %; ≥11 years: 35 %; p = 0.052) and frequency of injections in the last 1 month (no injection: 41 %; 1–10 days: 41 %; 11–20 days: 35 %; 21–30 days: 32 %; p < 0.004). Thus, the risk of HIV and HCV infection in our cohort was mainly concentrated around injection behaviors and not sexual behavior. Among the sexually active PWIDs, 70 % (586/837) reported unsafe sex in the last 3 months. Unsafe sex among PWIDs remains a key programmatic concern.

## Discussion

In this paper, we report a high prevalence of HIV infection (25.9 %), hepatitis C infection (53.7 %), and more significantly, high HIV-HCV co-infection (19.6 %) among male PWIDs in Delhi in 2012. HBV prevalence was lower at 9.7 %; while HIV-HBV co-infection was 3.4 % overall, among HIV positive PWIDs without HCV the prevalence was 26 %. Our findings are at variance with a study conducted among PWIDs in Delhi in 2003 [16], which reported a prevalence of 37 % for HIV, 36 % for HCV infection, HIV-HCV co-infection of 9.6 % and 40 % prevalence for HBV. The annual HIV Sentinel Surveillance (HSS) [22] has documented a change in HIV prevalence among PWIDs in Delhi from 14.4 % in 2003, 10 % in 2006 to 18.2 % in 2010–11. The HSS is an annual exercise to monitor trends in HIV prevalence; HIV testing is conducted among 250 PWIDs, recruited through snowball sampling at two DICs in Delhi. One DIC overlaps geographically with one of our study sites. Our HIV, HBV and HCV prevalence are similar to those reported more recently from Chennai [9] with a HIV prevalence of 29.8 %, HCV prevalence of 62.1 % and 11.1 % for HBV; and a recent study from Delhi that reports a HIV prevalence of 13.8 %; HCV mono-infection of 31 % and HIV-HCV co-infection of 14.5 % [23]. Another recent study from Punjab reports a similarly high prevalence of HIV (29 %), HCV prevalence of (49 %) and HIV-HCV co-infection 25.7 % among PWIDs [18].

Existing reports about HCV prevalence are based predominantly on serological testing for anti HCV antibodies, and a positive result can represent an acute, chronic or resolved HCV infection [8, 16, 18, 23–26]. We report the prevalence of active HCV infection based on HCV RNA PCR testing; to the best of our knowledge this is the first study in the country to provide these estimates for a large cohort of PWIDs. This has relevance for the national program as only PWIDs with active HCV infection need treatment. Our finding that a quarter of the PWID with a positive HCV antibody test were negative for HCV RNA, suggests that existing studies, which predominantly used antibody test to report HCV prevalence, overestimate infections substantially.

Several studies report a higher HCV prevalence among PWIDs than HIV prevalence [9, 18, 25, 26]. HCV is estimated to be 10 times more infectious than HIV, per unit of blood required for transmission [27] and recent evidence showing that HCV can remain infectious, after drying at room temperature, for up to six weeks in blood spots [28], may provide a biological explanation for the continuing spread of HCV among PWIDs as they continue to practice unsafe injection practices that include sharing equipment, drawing from a common container and injecting with used needles.

HBV prevalence was lower overall (9.7 %). The prevalence among the HIV positive PWIDs (12.8 %), including those with HCV infection, was similar to the 12 % reported by Solomon et al., from Chennai [8]. However, it is noteworthy that HBV prevalence among HIV-positive participants, in the absence of HCV, was 26 %. A study conducted in Manipur among HIV-positive PWIDs detected HB core antibodies, a marker of prior infection, in all their participants implying that all had been exposed to HBV in the past [14]. Most studies have used HBsAg as the marker of HBV [8, 16]; it is possible that in the presence of a high prevalence of active HCV infection, all HBV infections may not be detected using HBs antigen testing. HBsAg is a marker of acute infection that persists during chronic infection. However, in some patients with acute infection (1-5 %) and in the rare chronic low-level carrier, levels of HBsAg may be too low to be detected by standard assays and other tests may be required [29]. It is well documented that in dual infections, HBV and HCV interact, and affect immune responses. Available evidence demonstrates that the two viruses can inhibit each other simultaneously; the chronology of infection has a role in determining the dominant virus; and HBV and HCV can alternate their dominance [30–32]. However, the overall dominant effect in published literature appears to be HCV suppression of HBV. Furthermore, patients with chronic HCV may have occult or silent HBV infection with low levels of circulating HBV DNA, and lack the HBsAg and HBeAg markers and their antibodies [30, 33]. Undetected HBV is a concern as the virus can be transmitted through percutaneous, sexual and mother-to-child routes. Future studies should consider using a wider panel of tests to determine HBV prevalence in this high risk population, as a one-time HBsAg test may underestimate prevalence.

As HBV and HCV co-infected patients have a higher risk of progressing to fulminant hepatitis, developing chronic infection, cirrhosis and hepatocellular carcinoma [2, 11], it is extremely important for targeted intervention programs for PWIDs to include HBV and HCV in prevention messages and counselling protocols for PWIDs and their families. TI programs should include HBV testing and vaccination, an effective prevention intervention. As there is no vaccine available for HCV and it may be a while before treatment becomes affordable and available through public health programs, the focus of primary prevention efforts should continue to be on safe injection practices in this population.

Unsafe injection practices, duration of injection drug use and frequency of injections were the main predictors for HCV mono-infection and HIV-HCV co-infection. Accessing NSP services in the last 3 months was associated with a higher risk of HIV mono-, HCV mono- and HIV-HCV co-infections and having comprehensive HIV knowledge was associated with higher risk of HIV-HCV co-infection. These findings must be interpreted with caution. Further analysis showed that the use of NSP services and having HIV knowledge increased with longer duration of injection drug use, and higher frequency of injections. It is very likely that PWIDs who had been injecting for longer periods and were injecting more frequently were more aware of harm reduction programs in the area, had a greater need for needles/syringes and had been exposed to IEC activities in the area, and therefore, were accessing NSP services. At the same time, more than half the participants continued unsafe injection practices in the last one month, especially, those who injected more frequently and accessed NSP services. It is possible that they are unable to obtain a sufficient supply of needles to meet their need. Thus, the main risk of infection remains rooted in unsafe injection practices, longer duration of injection drug use and more frequent injections. In our baseline findings, HIV positive participants reported higher risk behaviors and accessed NSP services more often [20]. As this is a prevalence study we cannot comment on when the infections occurred; thus, it is also very likely that among long duration PWIDs the infections may have occurred before participants started accessing NSP services. The majority of our participants were not sexually active (63 % overall and 74.3 % among HIV-HCV co-infected participants) and sexual activity was not associated with increased HIV, HCV or HIV-HCV co-infection. However, among PWIDs who were sexually active, 70 % reported unsafe sex in the last 3 months, which remains an important concern for the HIV prevention program.

More than half our study participants had never consumed alcohol. This is in contrast to findings from a 2012 study on drug use patterns across 11 states that reports the majority of PWIDs ever used alcohol in their lifetime [34]. It is possible that our study participants transitioned to injection drug use from other addictions without consuming alcohol. In our previous study with 800 PWID in Delhi, only 23 % reported alcohol consumption [19]. Overall alcohol consumption in the general population in Delhi is low – 33.1 % of men 15–49 years of age interviewed in NFHS-3 reported consuming alcohol [35].

The study is not without limitations. Just two-thirds (66.2 %) of our cohort returned for this follow up visit; while this does not impact the reporting of prevalence for HIV, HBV and HCV infections, the program would have benefitted from prevalence reported from the larger cohort. We used only HBsAg testing to detect HBV infection. Using a wider panel of tests may have allowed us to detect occult HBV infections or past exposures; this may be considered for future studies. Further, we would have benefited from follow-up HBV and HCV testing at FV2, in this cohort of PWIDs, to obtain incidence rates that would have provided important information for the program; unfortunately we could not do so due to resource constraints. Despite using multiple strategies to reach female PWIDs including female seeds for peer-referral and female out-reach workers, we were able to recruit only 26 female PWIDs. In our previous study in 2006, we used respondent-driven sampling and were able to only recruit 17 female PWIDs [19]. It is very likely that there are few female PWIDs in Delhi. This finding has also been reported from other parts of India and in Asian countries [9, 36]. Anecdotal information from ORWs in Delhi suggests that non-injecting drug-use is more frequent among female drug users; further research with female non-injecting drug users is needed to better understand drug use in this population.

## Conclusion

In conclusion, we report a high prevalence of HIV, HCV and HIV-HCV co-infection among male PWIDs in Delhi. The prevalence of HBV, although lower than HCV, is still high with nearly a tenth of the PWIDs carrying the virus. The PWID population continues to engage in unsafe injection practices and not access NSP services regularly. Targeted intervention programs need to urgently address this problem by including hepatitis B and C in HIV prevention messages and counseling, expand services to include hepatitis testing, and offer hepatitis B vaccination for HBV negative PWIDs. A push by governments and activists to reduce the cost of treatment for hepatitis C is also needed to widen access to treatment for infected persons.

## Abbreviations

PWID: People Who Inject Drugs
HIV: Human Immunodeficiency Virus
HBV: Hepatitis B
HCV: Hepatitis C
NACO: National AIDS Control Organization (of India)
NSP: Needle/syringe Program
OST: Oral Substitution Therapy
RRR: Relative risk ratio
CI: Confidence interval
IQR: Inter-quartile range
